# Integrated, multidisciplinary care for hand eczema: design of a randomized controlled trial and cost-effectiveness study

**DOI:** 10.1186/1471-2458-9-438

**Published:** 2009-12-01

**Authors:** Robin F van Gils, Pieter GM van der Valk, Derk Bruynzeel, Pieter J Coenraads, Cécile RL Boot, Willem van Mechelen, Johannes R Anema

**Affiliations:** 1 Department of Public and Occupational Health and EMGO Institute for Health and Care Research, VU University Medical Center, Amsterdam, the Netherlands; 2 Department of dermatology, Radboud University Nijmegen, the Netherlands; 3 Department of dermatology, VU University Medical Center, Amsterdam, the Netherlands; 4 Department of Dermatology, Groningen University Medical Center, the Netherlands; 5 Body@Work, Research Center Physical Activity, Work and Health, TNO-VU University Medical Center, Amsterdam, the Netherlands; 6 Research Center for Insurance Medicine AMC-UWV-VU University Medical Center, Amsterdam, the Netherlands

## Abstract

**Background:**

The individual and societal burden of hand eczema is high. Literature indicates that moderate to severe hand eczema is a disease with a poor prognosis. Many patients are hampered in their daily activities, including work. High costs are related to high medical consumption, productivity loss and sick leave. Usual care is suboptimal, due to a lack of optimal instruction and coordination of care, and communication with the general practitioner/occupational physician and people involved at the workplace. Therefore, an integrated, multidisciplinary intervention involving a dermatologist, a care manager, a specialized nurse and a clinical occupational physician was developed. This paper describes the design of a study to investigate the effectiveness and cost-effectiveness of integrated care for hand eczema by a multidisciplinary team, coordinated by a care manager, consisting of instruction on avoiding relevant contact factors, both in the occupational and in the private environment, optimal skin care and treatment, compared to usual, dermatologist-led care.

**Methods:**

The study is a multicentre, randomized, controlled trial with an economic evaluation alongside. The study population consists of patients with chronic, moderate to severe hand eczema, who visit an outpatient clinic of one of the participating 5 (three university and two general) hospitals. Integrated, multidisciplinary care, coordinated by a care manager, including allergo-dermatological evaluation by a dermatologist, occupational intervention by a clinical occupational physician, and counselling by a specialized nurse on optimizing topical treatment and skin care will be compared with usual care by a dermatologist. The primary outcome measure is the cumulative difference in reduction of the clinical severity score HECSI between the groups. Secondary outcome measures are the patient's global assessment, specific quality of life with regard to the hands, generic quality of life, sick leave and patient satisfaction. An economic evaluation will be conducted alongside the RCT. Direct and indirect costs will be measured. Outcome measures will be assessed at baseline and after 4, 12, 26 and 52 weeks. All statistical analyses will be performed on the intention-to-treat principle. In addition, per protocol analyses will be carried out.

**Discussion:**

To improve societal participation of patients with moderate to severe hand eczema, an integrated care intervention was developed involving both person-related and environmental factors. Such integrated care is expected to improve the patients' clinical signs, quality of life and to reduce sick leave and medical costs. Results will become available in 2011.

## Background

The individual and societal burden of hand eczema is high. Hand eczema is a prevalent disease according to long-term registrations in general practitioner's-practices. Prevalence ranges from 25 to 66 cases per 1000 patient years. Point prevalence varies from 5 to 10% and incidence rates from 4 to 7% [1,2]. Self-reported life-time prevalence comes to more than 30% of the respondents. Hand eczema accounts for 90% of the occupational skin diseases and is in the top three of registered work-related disorders [3]. Literature also indicates that hand eczema is a disease with an unfavourable prognosis. The pathomechanism of hand eczema is often multifactorial. Both endogenous and exogenous factors play a role in its pathomechanism. Even meticulous avoidance of contact factors does not grant cure. Less than 50% of the patients has been reported to be cured after 5 years [4].

Significant numbers of patients suffer from this disease and are hampered in their daily activities and work. The physical and psychosocial burden for patients with skin diseases is comparable to patients with other chronic diseases, like multiple sclerosis and migraine, and even higher than patients with diabetes mellitus. In 18% of the patients symptoms of a clinical depression is present [5]. Medical consumption is also high; 60% visit their general practitioner in a year and 20% visit a medical specialist. High costs are also related to productivity loss and sick leave. In the Netherlands, annual costs of medical care, absenteeism and disability pensions due to occupational skin disease in employees in 2001 were estimated at € 98,1 million [6].

Although the individual and economic burden is high, the current usual care by dermatologists is thought to be suboptimal. Coordination of care is essential to ensure optimal avoidance of relevant contact factors at home and in the working environment. Usual care does not include consultation with the general practitioner or occupational physician, and the people involved in the workplace. Hence, suboptimal care for patients with occupational skin disease may lead to long-term absenteeism and ultimately to permanent disability with unnecessarily high costs [7]. Patients are in need of extensive counselling to improve their situation, as hand eczema has a complex aetiology and runs a dynamic but chronic course. Patients need instruction and information on topical therapy, aggravating factors and preventive measures. Usual care cannot provide for this, because of the limited time and expertise available at most outpatient clinics [8,9].

Recent data indicate a better prognosis due to secondary prevention and protocolled intervention. A recent systematic review indicated that usual care for eczema can be improved by active care management by a nurse practitioner, with doctors often lacking the time to offer sufficient patient education, and to manage complexly organized care of chronic illnesses effectively[10]. This perspective prompted us to start with integrated, multidisciplinary care for hand eczema patients, aiming at optimal topical treatment, avoiding relevant contact factors at home and in the working environment as much as possible, and optimal compliance to proper skin care instruction. Based on available data and this systematic review, we hypothesize that integrated, coordinated multidisciplinary care will lead to improved clinical signs of hand eczema and quality of life, and a reduction in absenteeism from work.

### Objective

In this paper, we describe the design of a randomized controlled trial (RCT) comparing integrated care for hand eczema by a multidisciplinary team, with usual care by a dermatologist. The multidisciplinary team involves a dermatologist, a specialized nurse and a clinical occupational physician. The specialized nurse also serves the role of care manager, and is responsible for the coordination of the integrated care. The primary research question is 'Is integrated care for hand eczema by a multidisciplinary team effective and cost-effective, compared to usual care?'

## Methods

### Design of the study

**Figure 1:**
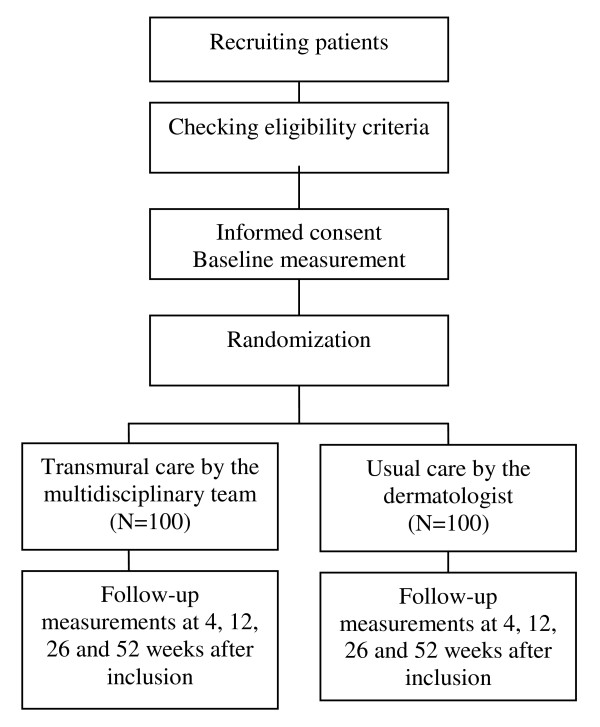
**Design of the RCT**.

The design of the study is a randomized controlled trial with a full economic evaluation alongside, with a follow-up of one year. The design is presented in figure 1. The Medical Ethics Committees of the participating hospitals (the VU University Medical Centre, Radboud University Medical Centre, Groningen University Medical Centre, Canisius Wilhelmina Medical Centre and Jeroen Bosch Medical Centre) approved the study. All participants will sign an informed consent. Exemption from insurance was granted by the Medical Ethics Committee of the VU University Medical Centre.

### Participants

The population consists of patients (16 years and older) with chronic (> 3 months) hand eczema who visit a dermatologist of one of the participating hospitals. The degree of hand eczema is determined using the Photographic Guide [11]. Patients with moderate to severe hand eczema are eligible. Patients with mild hand eczema who are on sick leave from work, or who score at least 4 points on a Visual Analogue Scale (VAS) for perceived burden of disease in the last three months before inclusion are eligible also. Exclusion of patients occurs in case of 1) generalized eczema, where hand eczema is not the main disease; 2) use of topical pharmacotherapy or phototherapy, other than used in the study; 3) systemic treatment affecting hand eczema; 4) inability to complete questionnaires written in Dutch language. Patients who meet the criteria will be given the patient information letter by their dermatologist and will be contacted by the researchers by telephone. In this contact, the researcher provides additional information about the consequences of participation. If the patient continues to be willing to participate, the researchers plan a face-to-face appointment to sign the informed consent form, to obtain baseline measurements and to perform the randomization procedure. Patients will be asked to fill out a form to allow their general practitioner or occupational physician, to provide information regarding medication and other relevant medical information to the multidisciplinary team.

### Randomization

Patients will be randomized by the research assistant using sealed opaque envelopes. Randomization will be performed at individual patient level. Stratification will be applied for: 1. Medical centre, 2. Risk profession and/or activities, according to the guideline Contact Eczema [12]. Random length blocks will be generated for each stratum by means of a computer-generated random sequence table. An independent statistician will prepare sequentially numbered sealed envelopes containing leaves that indicate the intervention group or the control group. Treatment allocation will take place after informed consent and completion of baseline measurements. After randomization, the outcome of the randomization will be reported by the research assistant to the patient and the dermatologist.

### Blinding

Clinical scoring of the primary outcome measure will be performed by an independent trained clinical investigator, who will be blinded for allocated treatment. The therapists of the multidisciplinary team will not be involved in assessing any of the outcomes. Participants and health professionals cannot be blinded for the allocated treatment. Because the questionnaires will be sent to the patient by mail, researchers and care providers are not likely to influence the way patients complete the questionnaires. After randomization, all participants will receive a non traceable research code consisting of a consecutive number. A research assistant will enter all data in the computer by the research code. Therefore, the analyses of the data by the researchers will be blind.

### Interventions

#### Integrated care by the multidisciplinary team

The group of patients randomized to the integrated care for hand eczema in a specialized centre will receive coordinated care by a multidisciplinary team. The multidisciplinary team consists of a care manager (an in dermatology specialized physician assistant or nurse), a dermatologist, an occupational physician and a specialized nurse. All professionals will be trained in the study protocol prior to the study, with a refresher course after a half year. The specific role of each member is described below.

##### 1. The care manager: coordination and communication

The care manager coordinates the integrated care. He/she is responsible for communication of the treatment plan with the patient. In addition, the care manager is responsible for the communication with all stakeholders, in the hospital (dermato-allergologist, occupational physician) as well as the relevant stakeholders in primary care (the patient's general practitioner and if applicable occupational physician and occupational hygienist). The care manager will have an intermediary role between primary and secondary care.

##### 2. Multidisciplinary team: discussion

All patients will be discussed weekly in the multidisciplinary team. The outcomes of this discussion will be the guideline for further treatment.

##### 3. Dermatologist: clinical evaluation

The dermatologist will perform the clinical and allergo-dermatological evaluation. This includes thorough history taking (including relevant exposures), physical examination and allergological testing. In addition to the standard allergological testing (Intracutaneous test with a small series of aero-allergens and/or epicutaneous tests with the European baseline series [13]), and a routine additional series) as in usual care, series relevant for the situation of the patient at work and at home can be tested with dilution series and controls.

##### 4. Specialized nurse: topical treatment and education

**Figure 2:**
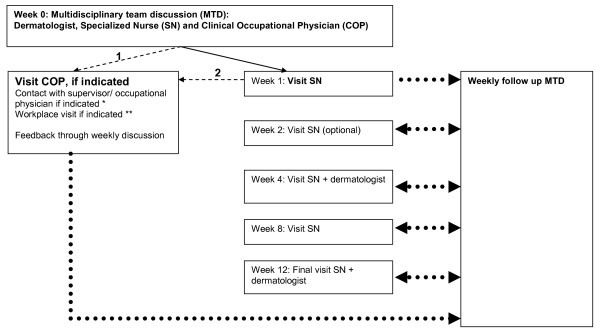
**Flowchart of the intervention**. 1: in case of work-related/occupational dermatitis or aggravated dermatitis, and/or in case of (potential) absenteeism. 2: in case of Incomplete task analysis, Unknown substances, Causal relation unknown earlier, (Potential) absenteeism or if Contact with supervisor is indicated. *: in case of (potential) absenteeism, incomplete task analysis, advices about modified/alternative work or supplementary material needed. **: in case of insufficient diagnostic information/supplementary material needed, insufficient information for proper treatment or care/prevention advice. **...**: information flow. __: referral; ---: referral if indicated.

All patients will visit the specialized nurse after 1, 4, 8 and 12 weeks. Another visit after 2 weeks is optional (figure 2). An essential part of the visits is education and counselling of the patient in the compliance to topical treatment. Topical treatment will be standardized. The approach is stepwise and strictly protocol led. Depending on the status of the hand eczema (acute, sub-acute or chronic lesions) and the overall severity (investigator's global assessment, [11]) a topical treatment regimen will be selected and adjusted during follow-up visits if needed. Treatment consists of dermatocorticosteroids and emollients, if needed supplemented with calcineurin-inhibitors.

Another important part of the programme is education of the patient, in order to improve his understanding of the mechanics of eczema and the circumstances that influence the barrier function of the skin. Instruction and counselling will be given regarding work, hand washing and care procedures, the use of protection measures such as protective gloves in general and the use of cotton gloves worn underneath. If necessary, information will be provided by the specialized nurse also for colleagues.

##### 5. Clinical occupational physician: information, instruction and workplace visit

The clinical occupational physician will be involved if hand eczema is work-related (occupational dermatitis or work-aggravated dermatitis), or when (potential) absenteeism as a result of hand eczema threatens (figure 2). Information will be gained about exposure to skin irritating circumstances and the use of protective measures. In addition to the standard allergo-dermatological testing (European baseline series and additional (occupational) series), material derived from the workplace can be tested. Workplace visits will be organised, if indicated, to gain relevant material for testing or information on work circumstances. The clinical occupational physician will also give advice about prevention and work procedures. If needed, provision of modified work will be organized in communication with the employer's supervisor. Contact with the employer's supervisor or occupational physician will take place if indicated.

#### Web-based patient tracking system

The process of the intervention will be recorded using a web-based patient tracking system. All professionals will receive a unique username and password. For each patient, data about each particular visit will be registered. Professionals will be alerted if an appointment has to be made. The care manager will be responsible for the input of data by all professionals. The web-based patient tracking system supports the professionals to deliver care according to the protocol.

#### Usual care

The patients who are allocated to the usual care group will receive allergological evaluation (intra-cutaneous tests and/or epicutaneous test with the European baseline series and additional series) delivered by the patient's own dermatologist, not working in a specialized centre. The patient's own dermatologist will also be responsible for further usual medical care, such as pharmacotherapy, provision of standard written information and advices. The patients' own general practitioner, occupational physician and treating medical specialist will be informed by letter about the study and the allocation of their patient to the usual care group. They will be asked to adhere to their normal treatment for hand eczema.

### Data collection

Clinical scores of hand eczema and self-reported questionnaires will be assessed by an independent and blinded clinical investigator at baseline and after 4, 12, 26 and 52 weeks. The clinical investigators will be trained in scoring methods prior to the study, with multiple refresher courses throughout the study. Self-reported questionnaires will be handed out and filled in at the same moments. Prognostic outcomes will be assessed at baseline. The direct and indirect medical costs will be measured monthly by means of cost calendars. Additional questions about the multidisciplinary care will be attached to the 12-week questionnaire of the first 30 patients randomized to the intervention group. A questionnaire about the patients' satisfaction with health care will be attached to the 12-week questionnaire of all patients.

### Outcome measures

#### Primary outcome

The primary outcome measure of the study is the cumulative difference in reduction of the clinical severity score HECSI (Hand Eczema Severity Index, [14]). The cumulative difference is defined as the area under the curve of the scores at baseline and after 4, 12, 26 and 52 weeks. The HECSI measures clinical severity of hand eczema on the following signs: 1. Erythema, 2. Infiltration/Papulation, 3. Vesicles, 4. Fissures, 5. Scaling and 6. Oedema. It will focus on the fingertips, the palm of the hand, the back of the hand and the wrists. For each area the intensity of each sign will be graded on the following scale: 0, no skin changes; 1, mild disease; 2, moderate and 3, severe. For each location, the affected area will be given a score from 0-4 (0 = 0%, 1 = 1-25%, 2 = 26-50%, 3 = 51-75% and 4 = 76-100%) for the extent of clinical symptoms. Finally, the score given for the extent for each location will be multiplied by the total sum of the intensity of each clinical sign. The total sum is called the HECSI score, varying from 0 to a maximum of 360 points.

#### Secondary outcomes

- Other clinical severity measures. Various symptoms will be scored using the Hand Eczema Area and Severity index (HEAS, [15]). This score is comparable to the HECSI in symptoms measured, but scores both hands separately.

- Disease-specific quality of life. The impact of hand eczema on the patient's quality of life is measured using a modified Dutch version of the Impact of chronic Skin Disease on daily Life (ISDL, [16]), in which the term 'skin disease' is replaced with 'hand eczema', and the Skindex [17]. The questionnaires discuss multiple aspects of hand eczema, such as impact on daily routine and work, and emotional and societal burden.

- Generic quality of life, using the standard Dutch version of the EuroQoL EQ-5D [18].

- Patients' global assessment, using a self-constructed Visual Analogue Scale (VAS).

- Total number of days of sick leave, using a monthly calendar.

- Direct medical costs are measured using cost calendars. The cost calendar includes direct costs relevant to the treatment of hand eczema, such as visits to the general practitioner, occupational physician or occupational hygienist and purchase of medication.

- Patient satisfaction will be measured with the Patient Satisfaction with Occupational Health services Questionnaire (PSOHQ, [19])

- Health care professionals' compliance to transmural care, advises and protection measures will be evaluated in a process evaluation.

#### Prognostic factors

Hand eczema is strongly related to wet and dirty activities or work. Working in a high risk job or with specific materials is considered to be prognostic for hand eczema. To determine risk profession, the guideline for contact eczema of the Dutch association for occupational health will be used [12].

### Sample size

The sample size calculation is based on a pilot study carried out in the Radboud University Medical Centre. In this pilot study, the Hand Eczema Area and Severity score (HEAS) was used. In the pilot study, a reduction in HEAS of 50% was observed during the first six months after the intervention. A reduction of this percentage to 40% during the next six months is hypothesized. The standard deviation (on a logarithmic scale) of the HEAS was 1.2 and the correlation between measurements from 1 to 6 months apart was 0.5. The correlation did not depend on the length of the interval. Based on these findings, a two-sided type 1 error of 5% and a power of 80%, 85 evaluable patients with at least three follow-up assessments are required per treatment group. Taking into account 30 dropouts, 200 patients will be enrolled, equally distributed over the centres.

### Co-interventions and compliance

During the intervention period co-interventions cannot always be avoided. In the intervention group, the patient's general practitioner will receive a letter, in which he/she is asked to leave the treatment of hand eczema to the multidisciplinary staff. Information about all treatments and co-interventions received by the patients will be collected by means of cost-calendars and questionnaires.

### Data analysis

All analyses will be performed at patient level. To examine the success of randomization, descriptive statistics will be used to compare the baseline measurements of the two groups. The primary analysis will be based on the intention-to-treat principle. For each patient, the area under the curve (AUC) of the HECSI scores over the period from 4 weeks to one year will be determined. An analysis of covariance will then be carried out with the logarithm of the AUC as dependent variable and treatment group, the logarithm of the baseline HEAS and the stratification factors of the randomisation as independent variables. In addition, per protocol analyses will be performed. In an exploratory analysis, other baseline covariates will be added to the model. Other endpoints will be analysed in the same way.

### Economic evaluation

Cost-effectiveness will be evaluated form a societal perspective. Monthly costs calendars will be used to measure direct and indirect medical costs and sick leave. The cost calendars will include direct health care costs relevant to the treatment of hand eczema, such as visits to a general practitioner or an occupational practitioner and self-bought medication or skin protection tools. Sick leave as a result of hand eczema will be registered monthly using the same calendar. Medical costs related to the study protocol will be obtained from the web-based tracking system, which keeps track of all visits to health care professionals and medication prescribed. Health care costs will be valued according to the prices suggested in the guidelines for economic evaluation in the Netherlands [20,21]. If cost-guidelines are not available, costs will be estimated using real prices. Costs of lost productivity caused by (partial) sick leave due to hand eczema will be calculated from the number of days of sick leave. Indirect costs will be calculated using the friction cost approach and the human capital approach. Quality-Adjusted Life Years (QALYs) will be computed in order to perform a cost-utility analysis for the two alternative treatments. Utilities will be based on the mean values for the two groups of patients at baseline and follow-up measurements. Costs will be summated for each individual patient. Bootstrapping will be used for pairwise comparison of the mean groups to calculate mean differences in direct, indirect and total costs between the two groups of patients. An incremental cost-effectiveness ratio (ICER) will be calculated by dividing the difference between the mean costs of the two groups by the difference in relative reduction (percentage point) between the two groups. A cost-utility analysis will also be conducted in which the incremental costs per QALY will be estimated. Reliability of the cost-effectiveness ratios will be graphically presented on a cost-effectiveness plane and in acceptability curves. To compare the results of the cost-effectiveness analysis with other conditions, general health status will be measured according to the Dutch version of the EuroQol EQ-5D [18].

## Discussion

This study protocol presents a randomized controlled trial to investigate the (cost-) effectiveness of integrated, multidisciplinary care for hand eczema. Usual care mainly focuses on improving clinical signs of hand eczema and does not have major focus on the patient's participation in society. Attention to societal participation is necessary to reduce the high costs related to productivity loss and high risk for long-term disability and accompanied physical and psychological burden for the patient. Therefore, an integrated care program has been developed.

### Theoretical basis

An integrated care program was developed based on the International Classification of Functioning, disability and health (ICF, [22]). In line with the ICF, this integrated care intervention not only aims to improve clinical signs, but it also focuses on increasing social participation. The ultimate goal of the intervention is to optimize the patient's quality of life and social functioning.

Integrated care is a system intervention where multiple systems are incorporated. First, the health system, where health care professionals cooperate to improve not only clinical signs, but also strive towards an increase in participation. This enables consensus about the treatment and the advice given. Next, the private and work systems are incorporated in the integrated care program. By focusing on personal factors to increase self-management, and by investigating the workplace, every actor in the environment is actively involved to reach a similar goal: improvement of participation. In order to achieve a systematic change in all systems, personal as well as environmental factors are addressed in the treatment.

Based on the biopsychosocial model [23], integrated care aims to achieve behavioural change in the patient, by means of counselling using a cognitive-behavioural approach. Thereby, the patients' coping with hand eczema improves and his self-management increases.

Besides that, integrated care aims to minimize the patients' exposure to causal factors, for example by using the appropriate protection measures. The home situation as well as the workplace are taken into account. If the patient cannot fulfil his daily work, the possibility of modified or alternative work will be discussed. If needed, the patient's relatives or colleagues and supervisors are included in the treatment.

### Impact of results

Besides an improvement of clinical outcomes, we expect an increase of the patient's quality of life. If the intervention shows to be effective, it could be implemented on a larger scale. By increasing the patient's self-management of hand eczema, we also expect a decrease of medical consumption and fewer episodes of sick leave in the long term.

Results will become available in 2011.

## Competing interests

The authors declare that they have no competing interests.

## Authors' contributions

DB and PJC participated in the design of the study. JRA and PGMV conceived of the study and participated in its design. WM supervised the study design. JRA and CRLB gave critical comments on the draft manuscript. RFG is responsible for the data collection and drafted the manuscript. All authors read and approved the final manuscript.

## Pre-publication history

The pre-publication history for this paper can be accessed here:

http://www.biomedcentral.com/1471-2458/9/438/prepub
